# Associations between village-level norms on marital age and marital choice outcomes among adolescent wives in rural Niger

**DOI:** 10.1016/j.ssmph.2020.100621

**Published:** 2020-06-25

**Authors:** Holly Baker Shakya, Jay Silverman, Kathryn M. Barker, Charlotte Lapsansky, Jennifer Yore, Sani Aliou, Mohamad I. Brooks, Anita Raj

**Affiliations:** a Center on Gender Equity and Health, University of California San Diego, USA; b UNICEF, USA; c Pathfinder International, NIGER; d Pathfinder USA, USA

**Keywords:** Social norms, Early marriage, Child marriage, Niger, Community norms, Marital choice

## Abstract

Social norms, the often unspoken rules that dictate behavior, are increasingly understood to play a role in child, early and forced marriage (CEFM) practices, but are less frequently examined in quantitative research on CEFM. No research on this topic has focused on Niger, despite the country having the highest prevalence of child marriage in the world. This study examines the associations of community and individual-level norms on marital age and marital choice with the outcomes of girls' age at marriage and choice in marriage. We used data from a family planning evaluation trial conducted in three districts within the Dosso region of Niger. Survey data were collected from adolescent wives and their husbands (N = 582) on demographics, normative beliefs regarding girls' age at marriage and marital choice, and among wives, age at marriage and engagement in marital choice. We developed our community-level norm variables by using the aggregate data from husbands' and wives' norms and wives' CEFM experiences. Using crude and adjusted regression models, we assessed the associations between our norms variables and our CEFM outcomes. In this context of very high prevalence of CEFM, we found that village-level norms related to marital choice, particularly the norms of men, are associated with younger age of girls at marriage. We also found that younger age of girls at marriage is positively associated with lower likelihood of their engagement in marital choice. Further, we find that village-level norms related to a later age of marriage and support for marital choice, as well as adolescent wives' perceptions of community norms related to a higher age of marriage, are associated with higher odds of a wife having had marital choice. These findings suggest the value of community level social norms change on CEFM in Niger, and the importance of focusing on child marriage and girls’ marital choice simultaneously given their interconnection.

## Highlights

• Niger has one of the highest rates of early marriage globally.
• We use data from rural Niger to test associations of social norms and early marriage.
• Girls' age of marriage was associated with men's community norms on marital choice.
• Girls' marital choice was associated with men's community norms around marital choice.
• Interventions combating early and forced marriage may need to target male norms.

## Introduction

Child, early and forced marriage (CEFM) is an internationally-recognized human rights violation that disproportionately affects women and girls globally (UNICEF-UNFPA, May 2019; United Nations High Commissioner on Human Rights, 2019). Child marriage is defined as any marriage where at least one of the parties is under 18 years of age (African Commission on Human and Peoples 'United Nations High Commissioner for Human Rights, 2017; Fenn, Edmeades, Lantos, Onovo, 2015), and is considered to be a form of forced marriage, given that children are not able to express full, free and informed consent (African Commission on Human and Peoples’, 2017; Fenn et al., 2015; United Nations High Commissioner on Human Rights, 2019). The practice of CEFM has been associated with a host of poor social and health outcomes including lower educational attainment, fewer economic opportunities, unintended and adolescent pregnancy, and increased risk of maternal and infant morbidity and mortality (Nour, 2009; Anita; Raj, 2010; K. G.; Santhya, 2011). Given the health and social risks associated with this practice, the elimination of child marriage by 2030 has been included among the internationally-recognized Sustainable Development Goals, which were adopted by more than 190 countries in 2015 (United Nations, 2016). In order to meet this goal, a clearer understanding of the mechanisms through which child marriage occurs is required. Social norms reinforcing CEFM have been hypothesized as underlying these practices and are thus a potential lever for change (Bicchieri et al., 2014; Fenn et al., 2015; Heise et al., 2019; Shakya et al., 2018; Steinhaus et al., 2019; Taylor et al., 2019; UNICEF-UNFPA, May 2019). Although initial research examining the associations between norms and practices of CEFM suggests significant associations, this research is still nascent and has been conducted in a limited number of countries. (B. Cislaghi et al., 2019; Holly B Shakya et al., 2018). We know of no research that has focused on this issue in Niger, which has the highest rate of girl child marriage in the world (Keeley & Little, 2017). While rates of early marriage have decreased in countries around the world over the past few decades (Jackson, 2012), the rate of early marriage has changed very little in Niger (Fenn et al., 2015).

Niger is one of a small number of nations in which child marriage continues to be legal for girls. Niger civil code forbids marriage below age 18 for boys, but only below age 15 for girls (UNFPA WCARO, 2017). Public perception also reflects this bias. A recent survey conducted in Zinder, one of the most populous regions in Niger, found that 80% of adults agreed boys should be married at 18 years or older, as compared to only 31% of adults agreeing girls should be married at age 18 or older (Regional Institute of Statistics, 2016). Half of adults felt girls should be married between the ages of 15 and 17 years, and 19% felt girls should be married between the ages of 10 and 14 years (Regional Institute of Statistics, 2016). By the age of 15, 28% of Nigerian girls are married, and by age 18, 76% are married (Institut National de la Statistique (INS) and ICF International, 2013). Prevalence of the practice varies throughout the country, with the median age at marriage ranging from 15.6 years in rural areas to 19.5 years in the capital city of Niamey (Institut National de la Statistique (INS) and ICF International, 2013).

Previous work suggests myriad and intersecting determinants of child marriage (Bicchieri et al., 2014; Fenn et al., 2015; Islam et al., 2016; UNICEF-UNFPA, May 2019). For example, studies from Africa and South Asia point to: traditions and gender-discriminatory norms rooted in patriarchal values and ideologies; the lack of educational and economic alternatives to child marriage; as well as exacerbating social factors such as poverty, economic instability, conflict and humanitarian crisis (Ministry of Population, 2016; Regional Institute of Statistics, 2016; Svanemyr et al., 2015; UNFPA & UNICEF, 2018). Research from India suggests that girls who marry young are less likely to have a say in the choice of who they marry (Santhya et al., 2010), and that in areas with lower gender equality, the age of marriage is more likely to be lower (Desai & Andrist, 2010). The association between gender norms and early marriage is complex, however, as the age of marriage can increase in response to other factors, while unequal gender norms may remain relatively stable (Desai & Andrist, 2010; Jackson, 2012). The United Nations High Commissioner for Human Rights as well as the UNICEF-UNFPA Joint Programme to Accelerate Action to End Child Marriage has provided a number of recommendations aimed at addressing CEFM. These range from system-level legislative and legal accountability measures, to increased engagement with community leaders and heads of household, to socio-cultural shifts in the norms that support child marriage and gender inequality (UNICEF, 2018; United Nations High Commissioner for Human Rights, 2017).

Social norms are the informal sets of rules derived from social systems that prescribe what behavior is expected, allowed, or sanctioned in particular circumstances (Mackie et al., 2014). Norms are hypothesized to shape behavior through both descriptive and injunctive norms (Bicchieri & Mercier, 2014, pp. 37–54; Cialdini et al., 1991; Fishbein & Ajzen, 1975; Mackie et al., 2014). Descriptive norms refer to perceptions of regular behaviors performed within a community and serve as an indication of what behaviors or actions are acceptable or “normal” in a given situation (Cialdini et al., 1991). Descriptive norms are optimally measured by asking people within a community their perceptions of how prevalent a certain behavior or practice is (Mackie et al., 2015). However in the case of observable behaviors, aggregating the behavior at the level of a socially relevant group, like a community, can serve as a proxy. For example, the degree to which a girl is involved in choosing whom she marries and the age at which she marries may indicate descriptive norms in a community surrounding marital choice and marital age, respectively. Injunctive norms, by contrast, are an individual's perceptions or beliefs of what others within the community approve or disapprove of, which in turn influence behaviors through pressures to conform (Cialdini et al., 1991; Fishbein & Ajzen, 2010). For example, in the context of child marriage in Niger, there may be an injunctive norm prescribing the age at which individuals in a community believe a girl ought to get married. This may be assessed by asking an individual the age at which most people in their community believe a woman should get married. Norms may conflict with personally-held attitudes. An individual may personally be opposed to child marriage, but engage in the practice within their own family out of a need to comply with social expectations or pressure.

The enforcement of social norms is hypothesized to occur through individually-perceived pressure to conform to the wishes of important others or referents (Cialdini et al., 1991; Fishbein & Ajzen, 2010). Just who these individuals are remains in question and varies with the behavioral situation (Ajzen & Fishbein, 1970). A key task when examining normative influence, then, is to identify the most valid grouping of referents. Ideally, in norms research, reference groups to assess descriptive norms would be identified through the use of discrete social network ties (Shakya et al., 2014, 2017), but in much health and development research, such data are lacking (Mackie et al., 2014). Instead, researchers looking for evidence of descriptive norms generate data with measures across more crude social units, in which social ties are inferred, such as residents of the same village or neighborhood (the concept behind DHS clusters) to determine whether there is inter-cluster variation. High levels of variation across these spatial units are viewed as evidence of variability in norms (Mackie et al., 2014).

In this study, we examine injunctive and descriptive social norms related to both early marriage and marital choice and whether these are associated with girls’ age at marriage and involvement in marital choice in the context of rural Niger. We consider injunctive norms as self-reported individual perceptions of what the community believes regarding when girls should marry and whether they should be involved in the selection of their groom, as reported by married girls themselves, as well as their husbands. We consider descriptive norms based on the aggregate reports of behaviors at the village-level, to provide insight into whether village-level descriptive norms are associated with behavior even after accounting for individual-level injunctive perceived norms. Findings from this work offer important insights into how social norms affect harmful traditional practices such as CEFM and the level of norms upon which to intervene to most effectively address CEFM.

## Methods

### Study setting

This study involves secondary analysis of data from an evaluation of a family planning intervention conducted between 2016 and 2018 with young married couples in 48 rural villages within the Dosso, Doutchi, and Loga districts in the Dosso region of Niger. From each of the three districts, 16 villages were randomly selected based on the following inclusion criteria: 1) having at least 1000 permanent inhabitants; 2) primarily Hausa or Zarma-speaking (the two major languages of Niger); and 3) no known recent intervention specifically around family planning or female empowerment with married adolescent wives or their husbands. Data were collected at two separate time points: baseline (Wave 1) and post-intervention, or one year after the baseline assessment (Wave 2). Primary outcomes, age at marriage and choice of marriage, preceded both data collection points for all respondents. Given the present study is designed to examine normative influence on CEFM rather than intervention effectiveness, data from both intervention and control villages are included in analyses.

### Participants

As part of the larger evaluation trial, willing and eligible couples were randomly selected (using a random number generator) from a list of all eligible married female adolescents provided by each village chief. Eligible participants were married girls aged 13–19 years and their husbands, fluent in Hausa or Zarma, and residing in the village where recruitment was taking place with no plans to move away in next 18 months or plans to travel for more than 6 months during that period. Of those randomly selected from the willing and eligible list, 88% participated in the Wave 1 survey (N = 1010). Equivalent numbers of couples were selected from each of the three districts. There were no significant differences in wife age, husband age, or time spent away from the village between those who did and did not participate. In Wave 2735 men of the original sample participated. With missing data on some measures, the analytic subsample is comprised of adolescent wife-husband dyads (N = 581) from whom there was data from both Wave 1 and Wave 2 surveys. While the outcome preceded the data collection, and therefore a longitudinal analysis was not possible, due to the nature of the data collection some variables were only available in either Wave 1 or Wave 2.

### Recruitment and data collection

Research assistants visited the randomly selected households and conducted a Household Recruitment Screener to confirm eligibility. If the household did not have an eligible couple, research staff recruited a randomly selected replacement in their place. Staff made up to three visits to each of the selected couples; if researchers could not reach the couple after three attempts, they dropped recruitment of the couple into the study. For couples reached for study, sex-matched trained research staff conducted surveys separately with the young women and their husbands.

Surveys were administered in a private location in the village, out of earshot of others and in a location the participant deemed private. Research staff conducted surveys in either the Hausa or Zarma language, depending on participant's language preference. The survey took approximately 40–60 min to complete and was administered using pre-programmed tablets. The staff member then uploaded the encrypted, de-identified data via a secure internet connection on a weekly basis. The data was compiled into dyadic husband/wife observations to be able to include measures from both wives and husbands in our analyses.

### Measures

This study assesses two outcome measures using data captured at Wave 1: age at marriage and women's report of marital choice. Age at marriage was assessed as a single continuous variable. Women's report of marital choice was assessed using an item that asked women, “Who had the greatest say with regard to arranging your marriage to your husband?” Response options were: 1: Respondent chose, 2: Respondent and husband chose each other, 3: Respondent with someone else chose, 4: Respondent's family chose, 5: Husband or his family chose respondent, 6: Someone else chose, 7: Joint decision not including respondent. Responses were coded as 1, 2, as 3 as women engaged in the marital decision making process, all others were coded as not engaged. Men were asked a parallel question, using the following responses: 1: Respondent, 2: Respondent and wife jointly, 3: Respondent with someone else, 4: Respondent's family, 5: Wife's family, 6: Someone else, 7: Joint decision not including respondent. Response 2 was coded as women engaged in marital decision-making. All other responses were as not engaged.

The primary independent variables of interest were individual attitudes, injunctive norms at the individual-level, injunctive norms at the village-level, and descriptive norms. Attitude and descriptive norms variables come from Wave 1, and the injunctive norms questions were added into Wave 2. Attitudes were assessed via items asked of both men and women on optimal age at marriage for girls. Participants reported their perceptions of optimal age for girls' marriage as a continuous variable. To assess the injunctive norm regarding age at marriage, both women and men were asked: “What age would people in your community say is the best age for a woman to get married?” Participants reported normative age as a continuous variable. To assess the injunctive norm regarding female involvement in marital choice, both women and men were asked whether they agree or disagree with the following statement: “People in my village expect that girls decide when and who to marry.” To ascertain village-level injunctive norms, the responses from the individual-level injunctive norms questions were aggregated from both female and male respondents. Descriptive norms related to girls' age at marriage and marital choice were assessed by aggregating both women's and men's reports of the following to the village-level: wife's marital choice (aggregate proportion by village) and wife's age at marriage (aggregate means by village).

To address known confounders, the following sociodemographic variables that are associated with age at marriage for both wives and their husbands were added including: age, spousal age difference, and a binary measure of any Quranic education. Number of wives and residence with extended family were also assessed. Women's and men's secular education were also included, categorized as no formal schooling, incomplete primary, completed primary, and any secondary education. Economic covariates included household wealth and food insecurity. Household wealth was assessed via the standard household assets list, summing each item that was reported in the home: a watch, a mobile phone, a bicycle, a motorbike or scooter, a car or truck, or an animal drawn cart. Food insecurity was assessed via a single item on whether the respondent or any member of the respondent's family went without eating for an entire day in the past 30 days due to a lack of food. Women were also asked if they had worked in the past 12 months. Finally, to assess the influence of a key interpersonal communication concept in social norms and behavior change, an additional control for whether or not a community health worker had visited the individual woman was included, and as well as an aggregate of whether or not a community health worker had visited the women in the village. These variables were included upon recommendation of experts in the field with local knowledge indicating that presence of community health workers may be associated with normative change. While there is not published evidence of this point from Niger, findings from studies conducted in other LMIC contexts highlight the role and importance of community health workers in introducing information regarding potential social and health harms of traditional practices and supporting normative change in these practices as related to marriage and family (Kok et al., 2015; McClendon et al., 2018; Taleb et al., 2015). Though certainly not representative of all underlying interpersonal communication strategies, the community health worker, a widely trusted source of information and normative influence in Niger, frequently plays a key role in behavior change interventions in the country, and therefore serves as a useful proxy for considering the possible influence of extant community engagement activities that may be taking place at the village and household level.

### Statistical approach

For both of our outcomes, age at marriage and women's report of marital choice, between-village variation was tested, using a −2 log likelihood ratio test in which we compared the −2 log likelihood of a null model against a multilevel model clustering on the village. For both outcomes, significant village-level clustering was found (not shown), so all models, bivariate, and multivariable, were run using multilevel modeling clustering on village. Both the mean and median number of couples in each village was 12 (SD: 3.95, range 3–22; inter-quartile range: 9–15). Bivariate analyses were first used, linear regression for age of marriage, and logistic regression for a woman's report of marital choice, to examine the relationship between attitudinal and normative exposure variables and our two outcomes. For variables significant at p < 0.10, separate multiple linear regression analyses were conducted to examine the demographic variables of age at marriage and separate multiple logistic regression analyses to examine marital choice at the individual level. For both age at marriage and marital choice, the attitudinal and normative exposure variables that were significant in the separate analyses were included in one full model. Continuous measures, including all village-level aggregates, were scaled in order to improve interpretability. To assess whether the presence of community health workers may confound those results, those variables were included in a second model. Treatment arm was controlled for in all analyses, though treatment was not expected to have any association with the norms of interest as these were not a focus of the study, and the assessed behaviors preceded engagement in the study. Finally, all models were tested for multicollinearity using the variance inflation factor test in the car package of R (Fox et al., 2007).

## Results

### Characteristics of the sample

Sample characteristics are shown in Table 1. Within the husband and adolescent wife dyads (N = 581), husbands were notably older than were their wives. The mean age of wives within the sample population was 17.3 years (SD = 1.5), while the mean age of husbands was 26.1 (SD = 5.7). Husbands were on average 8.7 years (SD = 5.4) older than their wives. The mean age of marriage for women was 14.1 years of age (SD = 1.9), and 16% of marriages were polygamous. Of the approximately 41% of women reporting working outside of the home, almost all were engaged in unpaid agricultural work. There is weak to moderate correlation between the female and male attitudinal and normative variables (range: 0.04 and 0.60). The strongest correlation (*r* = 0.60) was seen between the village-level measure of the ideal age of marriage.

**Table 1:**
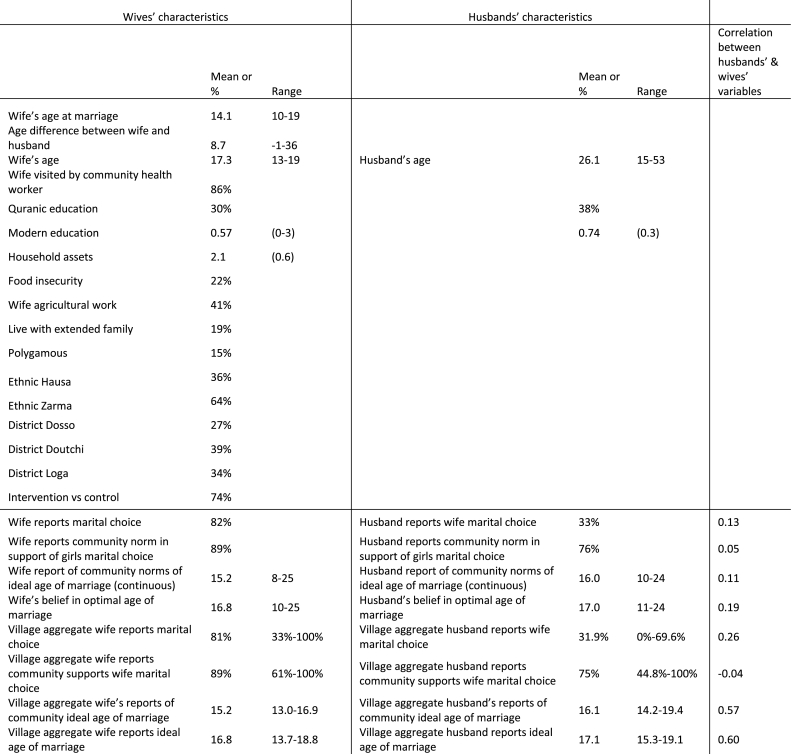
Sample characteristics. Observations are couple level dyads, N=581.

### Age at marriage

Table 2 shows the results of separate bivariate models for the outcome age of marriage and attitudinal or normative exposure variables. We found that not one of our individual attitudinal or normative exposure variables around marital choice and optimal age of marriage were significant, however all of our village-level aggregate measures were. Table 3 then shows the results of multivariable linear regression model, in which each village-level aggregate measures were used to predict age at marriage, with the inclusion of all sociodemographic controls. We found that the village aggregate of wife's report of involvement in marital choice was positively associated with a greater age of marriage, as was a higher village-level aggregate of wife's reported perception of the community's ideal age of marriage, village-level aggregate of husband's individual attitude of ideal age of marriage, and the village-level aggregate of the husbands perception of a community norm supporting wife's marital choice.

**Table 2:** Separate multi-level bivariate linear regression models showing the associations between individual and village norms and attitudes with age at marriage (stratified by female and male level variables) (N = 581 couple level dyads).

Variables reported by wives			Variables reported by husbands		
	Beta	P value		Beta	P value
Wife reports marital choice	0.08	0.27	Husband reports wife marital choice	0.11	0.13
Wife reports community norm in support of girls marital choice	0.05	0.54	Husband reports community norm in support of girls marital choice	0.03	0.70
Wife report of community norms of ideal age of marriage (continuous)	0.09	0.26	Husband report of community norms of ideal age of marriage (continuous)	0.05	0.47
Wife's belief in optimal age of marriage	−0.06	0.78	Husband's belief in optimal age of marriage	0.04	0.84
**Village aggregate wife reports marital choice**	**0.40**	**<0.001**	**Village aggregate husband reports wife marital choice**	**0.30**	**0.01**
**Village aggregate wife reports community supports wife marital choice**	**0.25**	**0.05**	**Village aggregate husband reports community supports wife marital choice**	**0.31**	**0.01**
**Village aggregate wife reports of community ideal age of marriage**	**0.32**	**0.01**	**Village aggregate husband's reports of ideal age of marriage**	**0.40**	**<0.001**
**Village aggregate wife reports ideal age of marriage**	**0.30**	**0.02**	**Village aggregate husband reports ideal age of marriage**	**0.33**	**0.01**

NB: variables in bold are statistically significant at the α = 0.10 threshold.

**Table 3:** Separate multi-level multivariable linear regression models showing the associations between individual and village norms and attitudes with age at marriage controlling for sociodemographics. (stratified by female and male level variables) (N = 581 couple level dyads).

Wives' Variables			Husbands' Variables		
	**Beta**	**P value**		**Beta**	**P value**
**Village aggregate wife reports marital choice**	**0.32**	**<0.001**	Village aggregate husband reports wife marital choice	−0.05	0.62
Village aggregate wife reports community supports wife marital choice	0.05	0.65	**Village aggregate husband reports community supports wife marital choice**	**0.26**	**<0.001**
**Village aggregate wife reports of community ideal age of marriage**	**0.17**	**0.08**	Village aggregate husband's reports of community ideal age of marriage	0.16	0.15
Village aggregate wife reports ideal age of marriage	0.02	0.88	**Village aggregate husband reports ideal age of marriage**	**0.18**	**0.10**

NB: variables in bold are statistically significant at the α = 0.10 threshold.

Results from the multivariable analysis (Table 4, Model 1), indicate that only village-level aggregate of wife's report of marital choice and village-level aggregate of husband's perception of a community expectation related to females being involved in marital choice remained significant. Normative variables retained significance when variables related to the presence of community health workers were added to the model (Table 4 Model 2). Every one standard deviation increase in the proportion of men in the village that report the community supports women's marital choice was associated with an increase in women's age of marriage of 0.22 years (95% CI 0.06–0.38). A one standard deviation increase in the proportion of women who report marital choice, was associated with an increase in women's age of marriage of 0.24 years (95% CI 0.01–0.42). We also found that community health workers seemed to be visiting women who have been married at a young age, and there is some evidence that they may be specifically working within communities where young age of marriage is the norm.

**Table 4:** Multilevel multivariable analysis showing the association with normative factors and age at marriage, controlling for sociodemographics (N = 581 couple level dyads, 47 village clusters).

	Null Model	Model 1			Model 2		
		Beta	SE	P	Beta	SE	P
Village aggregate wife reports of community ideal age of marriage		0.10	0.09	0.31			
Village aggregate husband reports ideal age of marriage		0.02	0.12	0.88			
**Village aggregate wife reports marital choice**		**0.21**	**0.11**	**0.05**	**0.24**	**0.10**	**0.04**
**Village aggregate husband reports community supports wife marital choice**		**0.17**	**0.09**	**0.06**	**0.22**	**0.08**	**0.01**
Proportion village visited by community health worker					−0.18	0.08	0.02
Wife visited by community health worker					−0.33	0.21	0.12
Age difference husband-wife		−0.41	0.08	<0.001	−0.37	0.08	<0.001
Quranic education husband		0.00	0.16	0.98	−0.05	0.16	0.75
Quranic education wife		0.02	0.18	0.93	0.03	0.18	0.87
Modern education wife		0.23	0.07	<0.001	0.24	0.07	<0.01
Modern education husband		0.07	0.07	0.33	0.09	0.07	0.24
Household assets		0.00	0.08	0.95	−0.04	0.07	0.55
Food insecurity		0.03	0.17	0.88	−0.03	0.17	0.84
Wife agricultural work		−0.37	0.19	0.05	−0.45	0.19	0.02
Live with extended family		0.20	0.18	0.26	0.22	0.18	0.22
Polygamous		1.00	0.23	<0.001	0.95	0.23	<0.001
Ethnic Hausa (ref)							
Ethnic Zarma		1.02	0.51	0.05	0.94	0.50	0.06
Ethnic other		1.15	1.29	0.37	1.42	1.28	0.27
Doutchi (ref Dosso)		0.86	0.54	0.12	0.73	0.52	0.25
Loga (ref Dosso)		0.54	0.21	0.01	0.39	0.21	0.07
Village proportion women agricultural work		−0.50	0.11	<0.001	−0.47	0.11	<0.001
Intervention vs control		−0.12	0.18	0.49	−0.15	0.18	0.38

AIC	2331	2285			2277		
ICC	0.15		0.002		0.000		

NB: variables in bold are statistically significant at the α = 0.10 threshold.

### Marital choice

Using the same analytic strategy as presented above, we first conducted a series of bivariate analyses looking at our individual and village-level attitudinal and normative exposure variables on the outcome of marital choice (Table 5). We found associations with several different factors, including the wife's perception of the community's ideal age of marriage, village-level aggregate wife's marital choice, and all village-level aggregate norms and attitudes reported by the husband. We next ran a series of separate multivariable models (Table 6) and found that after including covariates, our outcome of individual marital choice was associated with village-level aggregate women's reports of marital choice, wife reporting that the community supports an older age of marriage, husband reporting wife's marital choice, and the village-level aggregate of husband's perception that the community supports women's marital choice. In the final combined multivariable model (Table 7, Model 1), all of these factors retained significance. Again, after adding in individual visits from community health workers and village-level aggregate community health worker visits (Table 7, Model 2), we found no difference in the associations between our final normative exposure variables and marital choice, and a slight increase in the AIC suggests that the addition of those two variables does not positively contribute to model fit. The odds that a woman reports marital choice increased by 1.39 (95% CI 1.04–1.87) for every one standard deviation increase in the proportion of men in the community that believe the community supports marital choice. For every year increase in a woman's report of the ideal age of marriage within the community, the odds that she reported marital choice increases by 1.31 (95% CI 1.02–1.69).

**Table 5:** Separate multi-level bivariate logistic regression models showing the associations between marital choice and individual and village-level norms and attitudes (by sex) (N = 581 dyads, 47 village clusters).

Bivariate analyses: wives' variables			Bivariate analyses: husbands' variables		
	Beta	P Value		Beta	P Value
			**Husband reports wife marital choice**	**0.70**	**0.01**
Wife reports community norm in support of girls marital choice	0.08	0.49	Husband reports community norm in support of girls marital choice	0.07	0.56
**Wife reports of community norms of ideal age of marriage (continuous)**	**0.31**	**0.02**	Husband report of community norms of ideal age of marriage (continuous)	−0.06	0.62
Wife beliefs in optimal age of marriage	−0.25	0.42	Husband's belief in optimal age of marriage	−0.34	0.24
**Village aggregate wife reports marital choice**	**0.69**	**<0.001**	**Village aggregate husband reports wife marital choice**	**0.32**	**0.06**
Village aggregate wife reports community supports wife marital choice	−0.07	0.73	**Village aggregate husband reports community supports wife marital choice**	**0.50**	**<0.001**
**Village aggregate wife reports of community ideal age of marriage**	**0.32**	**0.06**	**Village aggregate husband's reports of ideal age of marriage**	**0.48**	**0.01**
Village aggregate wife reports ideal age of marriage	0.23	0.19	**Village aggregate husband reports ideal age of marriage**	**0.50**	**<0.001**

NB: variables in bold are statistically significant at the α = 0.10 threshold.

**Table 6:** Separate multi-level multivariable logistic regression models showing the associations between individual and village norms and wives reported decision regarding marriage (by sex) (N = 581 dyads, 47 village clusters).

Separate models wives' variables as primary predictor			Separate models husbands' variables as primary predictor		
	Beta	P value		Beta	P value
			**Husband reports wife marital choice**	**0.73**	**0.01**
**Wife report of community norms of ideal age of marriage (continuous)**	**0.24**	**0.06**	Husband report of community norms of ideal age of marriage (continuous)		
Wife's belief in optimal age of marriage			Husband's belief in optimal age of marriage	−0.34	0.24
**Village aggregate wife reports marital choice**	**0.61**	**<0.001**	Village aggregate husband reports wife marital choice	0.19	0.28
Village aggregate wife reports community supports wife marital choice	0.04	0.86	**Village aggregate husband reports community supports wife marital choice**	**0.42**	**0.01**
Village aggregate wifes reports of community ideal age of marriage	0.10	0.55	Village aggregate husband's reports of ideal age of marriage	0.28	0.19
Village aggregate wife reports ideal age of marriage	−0.20	0.40	Village aggregate husband reports ideal age of marriage	0.24	0.22

NB: variables in bold are statistically significant at the α = 0.10 threshold.

**Table 7:** Multilevel multivariable logistic regression analysis showing the association between individual and normative factors with women's reported decision regarding marriage (N = 581 dyads, 47 village clusters).

	Null Model	Model 1			Model 2		
		Beta	SE	P	Beta	SE	P
**Husband reports wife marital choice**		**0.84**	**0.29**	**<0.001**	**0.83**	**0.29**	**<0.001**
**Village aggregate wife reports marital choice**		**0.51**	**0.16**	**<0.001**	**0.52**	**0.16**	**<0.001**
**Village aggregate husband reports community supports wife marital choice**		**0.28**	**0.14**	**0.05**	**0.33**	**0.15**	**0.03**
**Wife report of community norms of ideal age of marriage (continuous)**		**0.24**	**0.13**	**0.06**	**0.27**	**0.13**	**0.04**
Proportion village visited by community health worker					−0.21	0.14	0.12
Wife visited by community health worker					0.22	0.35	0.54
Age difference husband-wife		−0.20	0.14	0.15	−0.19	0.14	0.17
Quranic education husband		0.27	0.28	0.32	0.23	0.28	0.40
Quranic education wife		−0.09	0.29	0.74	−0.13	0.29	0.66
Modern education wife		0.06	0.13	0.63	0.06	0.14	0.66
Modern education husband		0.03	0.14	0.84	0.05	0.14	0.70
Household assets		−0.08	0.12	0.51	−0.1	0.13	0.42
Food insecurity		0.04	0.30	0.9	0.03	0.30	0.91
Wife agricultural work		0.30	0.32	0.36	0.26	0.33	0.43
Live with extended family		−0.01	0.32	0.97	0.01	0.32	0.98
Polygamous		1.14	0.46	0.01	1.12	0.47	0.02
Ethnic Hausa (ref)							
Ethnic Zarma		−0.32	0.77	0.68	−0.33	0.77	0.67
Ethnic other		−2.49	1.64	0.13	−2.46	1.65	0.14
Doutchi (ref Dosso)		−0.08	0.82	0.92	−0.03	0.82	0.97
Loga (ref Dosso)		0.54	0.37	0.15	0.40	0.38	0.29
Village proportion women agricultural work		0.02	0.19	0.90	0.10	0.20	0.63
Intervention vs control		0.12	0.31	0.69	0.07	0.31	0.81

AIC	525.4	512.1				513.6	
ICC	0.19	0.00				0.00	

NB: variables in bold are statistically significant at the α = 0.10 threshold.

## Discussion

In this study, we use data collected from rural Niger to try to understand the relationship between marital choice and age at marriage, and the social norms specific to both. We find that norms around marital choice are those most strongly associated with the likelihood that an individual girl marries at an older age. We also find that norms around age at marriage and norms around marital choice are those most strongly associated with the likelihood that an individual girl reports marital choice. Our findings suggest that marital choice and age at marriage are strongly interconnected within these communities, and that both descriptive and injunctive norms may play an important role. These findings, the first of their kind from Niger, extend work largely from South Asia that highlights gendered restrictions against girls’ mate selection or even their perceptions of acceptability in choosing their spouse, and the strong association between early marriage and lack of marital choice among girls (Allendorf, 2017; McDougal et al., 2018), by demonstrating the importance of social norms in reinforcing and linking these practices.

Study findings also demonstrate that influential norms not only operate at the individual level in terms of perceptions but also at the community level, in terms of both practices and perceptions. In communities where a larger proportion of the girls report marital choice, and in communities where a larger proportion of the male population believes that the community supports marital choice, individual girls marry at older ages. These normative factors around marital choice are strongly associated with age at marriage whereas norms around age at marriage are not. Our analyses also indicate that when a greater proportion of women in a village believe that the community supports an older age of marriage, and when a greater proportion of men in the village believe that the community supports marital choice, girls in those communities are more likely to report having had marital choice. These findings support the potential value of community-level social norms interventions to address CEFM, and reinforce prior research on the importance of within-community efforts to promote normative change (Cislaghi & Heise, 2018). Importantly, prior research evaluating prevention of child marriage largely demonstrate the value of girl education and cash transfer programs as being most effective in altering the practice. We could identify no rigorous evaluation of normative change approaches for prevention of CEFM; this may be an important area ripe for study (Kalamar et al., 2016).

Our findings indicate a nuanced dynamic, as we note sex differences in the nature of normative influences associated with our outcomes of interest. Specifically, we find that the community level norms that are associated with both of our outcomes are the aggregate measure of men's perceptions of whether the community supports marital choice - an injunctive norm, and the aggregate of women's reported marital choice - a descriptive norm. These findings suggest that the community context is salient in different ways. What men believe the community supports around marital choice is strongly associated with both outcomes, while what women actually report in terms of their own marital choice is also strongly associated with those same outcomes. This is to our knowledge the first study that has quantitatively analyzed associations between norms and practices related to marital choice in Niger or elsewhere, as well as the first study that has considered sex differences in community-level social norms affecting traditional practices. Further research is needed to understand how these sex differences in normative effects may play out in different national contexts and as related to different outcome behaviors.

Our measure of the descriptive norm, the aggregate of women's reported choice, is a proxy for descriptive norms, as we did not ask women what they thought was taking place in their community. Furthermore, the fact that 82% of women report participating in the choice of their marriage, while only 32% of men report their wives participating in that choice suggests that choice in this context may be open to interpretation. Does choice mean actively identifying a potential husband; does it mean having a veto power; does it mean it was discussed with her but she was given little room but to acquiesce? The details of choice in this context are still unclear, consistent with varying interpretations of how marital choice is conceptualized in different Islamic communities (Relief, 2018; Riaz, 2013). Nevertheless, what is salient is that in community contexts where women interpret themselves as having had a choice in whom they married (i.e., communities in which descriptive norms indicate the practice of women's marital choice), individual girls within these communities are more likely to report having a choice of who they marry and to have married at an older age. Because this measure is an aggregate of women's reported choice, we also don't know whether they believe that other women also have made their own choice. It is possible that this measure is more likely giving us information about contexts in which women are more likely to believe that they have had a choice, rather than an objective measure of community behavior.

The association between the male perception of the community's support of marital choice and both of our outcomes is an important reflection of men's power in the domains of family, marriage, and fertility in this setting. While this offers an important leverage point for intervention, that same opportunity comes with its own risks. Our results suggest that engaging with men to change norms around marital choice may be an important strategy for shifting the dynamics of forced and early marriage in these settings. The caveat is, however, that targeting men in this way does not alter the patriarchal nature of these practices and in fact can reinforce male control over girls' marital practices unless the program specifically works to increase girls agency and change gender norms. At the same time, it can be difficult to engage women who were not given a choice in their marriage and who married as minors because the focus of the interventions is to change behaviors in the community that cannot be changed in their own lives, having already occurred. It is crucial to approach the issue with sensitivity in order not to stigmatize those for whom early or forced marriage has already occurred. These issues speak to the need for nuance in application of these findings to the field, with consideration of culture and context as well as ensuring an intersectional equity lens in the approach.

While these findings offer important insight regarding the influence of village-level norms, less clear is the value of the normative beliefs of individuals and their associations with CEFM. As noted above, wives' and husbands' normative beliefs at the individual level were not associated with wife's age at marriage after accounting for community norms. Wife's and husband's individual attitudes about appropriate age at marriage were also not associated with wife's age at marriage. However, wife's beliefs regarding community norms related to appropriate age of marriage for girls was associated with whether or not the wife reported marital choice herself. While these findings are captured at the individual level, they only reinforce the role community norms and expectations have on girls with regard to their marital choice. Consequently, alteration of these norms at the community level is important. However, these findings beg the question of how girls may or may not resist harmful practices of CEFM, which have been linked to increased maternal mortality in Niger (Institut National de la Statistique (INS) and ICF International, 2013; Verguet et al., 2016), and the familial and social consequences of this resistance. More research is needed to ensure that supporting girls' resistance and marital choice can be approached in ways that do not result in backlash against girls failing to adhere to social norms.

While our findings offer important insight into issues of social norms and CEFM in the context of a high need an understudied nation, Niger, they should be considered in light of certain study limitations. First, certain factors were associated with retention in the study between Waves 1 and 2. Adolescent wives were more likely to be missing Wave 2 data if at Wave 1 they were nulliparous (p = 0.02) or if their husband was polygamous (p = 0.06). Husbands were more likely to be missing Wave 2 data if at Wave 1 they were 15–24 years of age compared to older age (p = 0.09), if their wife had no schooling (p = 0.01), and if they had spent more than three months away from the village in the past year (p < 0.001). Data rely on self-report and thus are vulnerable to recall and social desirability biases. We do not anticipate much concern related to recall given the young age of the sample and thus recency of marriage, and given that age and choice at marriage are memorable phenomena. However, validity of age data may be questionable as access to birth registry data was not possible, and age, both at time of interview and at time of marriage, may not be precise in this population. However, again, given the young age of the population, we expect this study yields more accurate age data than larger scale studies such as the Demographic and Health Survey in Niger (Institut National de la Statistique (INS) and ICF International, 2013), which lack any data on social norms. While optimally descriptive norms would be measured by asking individuals their perceptions of community behaviors, lacking such measures we used a proxy measures of community level behavioral aggregates of individual behaviors.

Generalizability of the findings are also somewhat limited, as the sample was in three districts within the Dosso region of Niger, was specific to married girls involved in a family planning intervention trial, and was only able to include the sample retained for follow-up in that trial. Nonetheless, given the paucity of data from Niger and complex sampling used in the study site areas, these findings offer an important sample of married girls in Niger not seen in previous published research. Additionally and relatedly, the analyses are cross-sectional in nature and interpretation, impeding assumptions of causality from these findings. Of note, some norms data were only available in the follow-up data set, and thus we used norms variables from two points in time, with outcomes assessed at Wave 1. Ideally, in cross-sectional analyses all data would be taken at the same time point. However, we wanted to include the most comprehensive set of measures available given the novelty of the work on social norms and CEFM, and the increasing level of interest in norms as a lever for change (Darmstadt et al., 2019). Importantly, norms variables across the two waves of data were significantly associated as would be hypothesized, increasing our comfort in including all norms data for study.

An additional concern is that as the survey was not comprehensively designed to understand CEFM norms and practices in Niger and thus potential confounders, such as those related to agency, assets such as information access, and opportunities such as financial inclusion indicators, are not able to be included in our analyses. Additionally, understanding related to the measurement of both social norms and to the measurement of forced marriage are fairly new to quantitative research so there are not standard measures we could use for these assessments. Norms and marital choice measures were developed for this study, often adapted from prior work from our team, who include social and behavioral science experts on these topics and the study of measurement. These measures were built on deep conceptual understanding and prior testing in other national contexts both in the case of social norms and marital choice (Cislaghi et al., 2019; Mackie et al., 2014; Raj et al., 2014), as well as expert and field input on the questions prior to field testing them via cognitive interviews. We then implemented them in the field. Hence, while standard measures could not be used, we engaged in a rigorous process of measurement development to offer potential new measures on these topics.

## Conclusion

This study analyzed community and individual level norms related to early marriage of girls and girls' marital choice in rural Niger, with a sample of adolescent wives and their husbands. In this context of very high rates of child and early marriage we found that village-level norms related to marital choice, particularly the norms of men, may be a key driver of child and early marriage. In addition, earlier age at marriage for girls in this context is significantly associated with lower likelihood of their engagement in marital choice, a finding that may point to limited female empowerment as a driver of both. Further, we find that village-level norms related to early marriage and marital choice, as well as adolescent wives' perceptions of community norms related to early marriage, are associated with odds of a wife having had marital choice. Importantly, we did not find any association with men's or women's attitudes regarding appropriate age of marriage and wife's age of marriage or wifes report of marital choice. These findings suggest the value of community level social norms change on CEFM in this context, particularly targeting males, and suggest that approaches the focus on individual attitudes may not be effective. At the same time, such efforts will require care not to reinforce norm changes on acceptability of CEFM practices that are predicated on maintained male control over and sanctioning of these approaches. Further research is needed to consider how to simultaneously address these norms and reinforce women and girls' autonomy, agency and safety with regard to marriage, given findings of high risk for spousal violence and maternal mortality in the region (Institut National de la Statistique (INS) and ICF International, 2013; Kidman, 2017; Verguet et al., 2016). Gender transformative interventions addressing the intersection of social and gender norms underlying these practices may be useful and have shown success in other national contexts (Hay et al., 2019; Heymann et al., 2019). These findings suggest that community-level norms related to girls' marital choice and agency should be targets of interventions to help avert early marriage of girls in Niger. Such findings highlight that, while previous research has found that education certainly has value in helping delay marriage of girls in contexts affected by the practice (Kalamar et al., 2016), addressing social norms related to marital choice in conjunction with promoting girls education may be more impactful.

## Ethics and approval and consent to participate

This study was approved by the University of California San Diego IRB, protocol number #160407S and Niger Ministry of Health. Wives aged 13–17 years were included the study. Based on these individuals being married, they are not viewed as children in Niger and have rights to consent to participate in research and to receive family planning services without consent of their parents (i.e., they are considered emancipated). Similar to California Emancipation of Minors Law (Family Code Section 700-7002), in which minors who have entered a valid marriage are legally emancipated, according to customary law in Niger, children become independent from their parents after they first marry. Niger law does not legally define the age of consent beyond the age at marriage. Niger is also a patrilocal culture, so adolescent wives do not live in the same residence or often village as their family or origin, thus, even if deemed appropriate, it would not be feasible to obtain parental consent in this context. All participants provided verbal consent, as the Hausa and Zarma languages are not in written form. The consent script was written in French and administered verbally in the Hausa or Zarma language, based on the native language of the participant. The consent script was approved by the Niger Ministry of Health's Ethics Committee to conform to local laws and standards. In all cases, consent scripts and forms were also approved by University of California San Diego, IRB.

## Author statement

Holly B. Shakya conceptualization, methodology, formal analysis, writing original draft and review; Jay Silverman supervision, funding acquisition, investigation; Kathryn Barker writing original and review; Charlotte Lapsanky conceptualization, writing original and review; Jennifer Yore investigation and writing review; Sani Aliou investigation and writing review; Mohamad I. Brooks investigation and writing review; Anita Raj funding acquisition, supervision, conceptualization, writing orginal and review.

## Declaration of competing interest

The authors have no conflicts of interest or financial disclosures to report.
